# Dual‐Organelle Secretome Conjugation and Organ‐Uptake Tracking (DuO‐SCOUT) Enables Deep and Sensitive Mapping of the Secreted Proteins

**DOI:** 10.1002/advs.77190

**Published:** 2026-08-14

**Authors:** Fenglian Yang, Rui Qian, Jiaxing Song, Xingqi Meng, Jin Young Kim, Kwok On Lai, Li Wang, Liang Zhang

**Affiliations:** ^1^ Department of Biomedical Sciences College of Biomedicine City University of Hong Kong Hong Kong Hong Kong SAR China; ^2^ Biotech and Health Centre Shenzhen Research Institute of City University of Hong Kong Shenzhen Guangdong China; ^3^ Department of Neuroscience College of Biomedicine City University of Hong Kong Hong Kong Hong Kong SAR China

**Keywords:** adipose, BioID, leptin, obesity, secretome

## Abstract

Inter‐organ communication is governed by a complex “secretome,” yet mapping the journey of these factors from their origin to precise cellular destinations remains a fundamental challenge. Proximity labeling emerges as a powerful tool to dissect the secretome, yet conventional workflows typically rely on single‐compartment biotinylation at the endoplasmic reticulum (ER), failing to capture proteins that utilize unconventional secretion. Here, we present DuO‐SCOUT (Dual‐Organelle Secretome Conjugation and Organ‐Uptake Tracking), a high‐performance platform that simultaneously targets BioID2 to the ER and the trans‐Golgi network (TGN). This integrated strategy captures the full secretory maturation relay, increasing protein identification by over 120% compared to traditional ER‐anchored methods. We translated this in vivo using an Adipoq‐Cre mouse model to map the adipose secretome. To bridge the gap between systemic transport and tissue‐specific uptake, DuO‐SCOUT integrates BSPA (Biotin‐Specific Proximity Amplification), a visualization toolkit detecting biotinylated proteins with sub‐nanomolar sensitivity. In obese mice, we identified the piriform cortex (PIR) as a previously unrecognized extra‐hypothalamic sink for adipose‐derived leptin. This is associated with a localized neuroinflammatory signature, including significant induction of Il6. DuO‐SCOUT establishes a broadly applicable framework for dissecting the complex molecular logic of systemic organ‐organ communication.

## Graphical abstract

A novel dual‐organelle proximity labeling platform, DuO‐SCOUT, decodes the complex landscape of systemic organ‐organ communication by capturing both classical and unconventional secretomes. Application in metabolic models maps the adipose‐to‐brain secretory relay, identifying selective extra‐hypothalamic sites for adipose‐derived factors and expanding the molecular logic governing metabolic homeostasis and neuroinflammation.

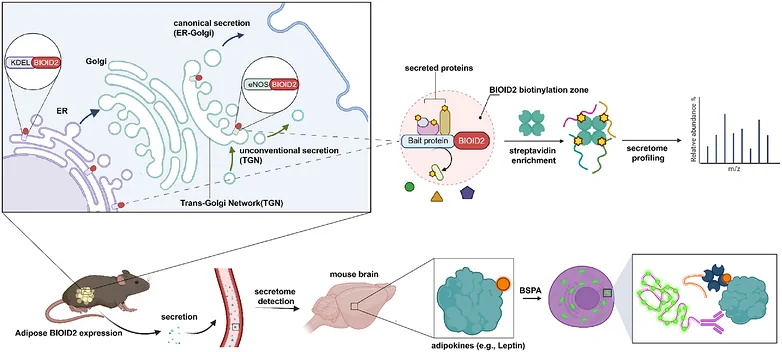

## 1 Introduction

Endocrine homeostasis is mediated by a complex and dynamic “secretome”—a diverse array of secreted proteins, peptides, and metabolites that facilitate essential inter‐organ communication [1, 2]. In the context of metabolic regulation, adipose tissue has moved beyond its classical description as a passive energy storage depot to emerge as a critical endocrine hub. It secretes an expansive repertoire of adipokines—such as leptin, adiponectin, and various signaling cytokines—that coordinately regulate energy expenditure, insulin sensitivity, and even higher‐order cognitive functions [3, 4, 5]. Recent studies have suggested that the adipose‐brain axis is not merely a feedback loop for satiety but a complex regulatory network that influences neuroinflammation and synaptic plasticity [4, 6]. However, identifying the full breadth of the in vivo secretome and, more importantly, tracking the precise spatial destinations of these factors within recipient tissues remains a formidable challenge in modern biology [7]. Traditional methods, such as systemic blood sampling and ex vivo explant cultures, provide only a “snapshot” of circulating levels. These approaches often fail to capture the actual secretory flux from specific depots or lack the sensitivity to detect key factors once they undergo massive dilution in the systemic circulation [7, 8, 9].

Proximity‐dependent biotinylation techniques, particularly those utilizing the BioID system [10, 11], have revolutionized our ability to map localized proteomes in living cells with high spatial fidelity [12]. When applied to the secretory pathway, common strategies typically anchor BioID in the endoplasmic reticulum (ER) lumen to biotinylate proteins that contain a signal sequence and go through the conventional ER‐Golgi secretion pathway [13, 14, 15, 16]. However, eukaryotic cells also have unconventional modes of secretion for proteins that lack a classical signal sequence for ER entry [17, 18, 19]. Such factors include hormones and cytokines that are important for endocrine homeostasis. For example, Tian et al. have identified that more than 60% of adipokines display no known signal sequence [20]. Recent studies have defined novel compartments that collect and sort unconventionally secreted proteins, including extracellular vesicles and a modified trans‐Golgi network [21]. Such knowledge suggests that single‐compartment biotinylation at the ER is insufficient for comprehensive profiling of the secretome, missing unconventional proteins that bypass the ER. Furthermore, even after successful biotinylation, a secondary bottleneck exists: once biotinylated factors enter the bloodstream, they are subjected to such extreme dilution that their site‐specific uptake in distant, blood‐brain barrier (BBB)‐protected organs like the brain becomes nearly invisible to conventional methods like immunohistochemistry or standard mass spectrometry [9].

To address these challenges, we developed DuO‐SCOUT (Dual‐Organelle Secretome Conjugation and Organ‐Uptake Tracking) that synergistically integrates a dual‐anchoring biotinylation strategy for profiling the secretome with an ultrasensitive proximity‐ligation assay (PLA) for tracking secreted factors [22, 23]. By simultaneously targeting BioID2 to the ER (via KDEL) and the Golgi (via eNOS) [24], DuO‐SCOUT captures a significantly expanded landscape of the secretome, increasing unique protein identification by more than 120% compared to the conventional ER‐labeling method. We subsequently translated this technology in vivo by engineering an Adipoq‐Cre‐driven DuO‐SCOUT mouse model [25], enabling high‐fidelity, tissue‐specific conjugation of the adipose secretome in a living physiological system. To bridge the gap between secretion and destination, DuO‐SCOUT coupled the proteomic profiling depth with BSPA (Biotin‐Specific Proximity Amplification), a proximity‐ligation‐based visualization toolkit designed to detect biotinylated traveling proteins within recipient tissues with sub‐nanomolar sensitivity [22, 23]. Applying the DuO‐SCOUT pipeline to a mouse model of obesity, we not only decoded the obesity‐induced shift in the adipose secretome but also discovered novel leptin‐associated proinflammatory activities in specific brain regions. We propose DuO‐SCOUT as a robust framework for dissecting the secreted proteome in organ‐organ communications.

## 2 Results

### 2.1 TGN‐Anchored Biotinylation Complements Conventional Secretome Profiling

Conventional secretome profiling typically utilizes proximity‐labeling enzymes anchored within the ER lumen, a strategy limited to proteins harboring classical signal sequences for the canonical ER‐Golgi secretory pathway [15, 16]. However, many critical hormones and cytokines—including 60% of adipokines—lack traditional signal sequences and bypass the ER via unconventional secretion modes [17]. Emerging studies have identified specialized compartments, including modified trans‐Golgi networks (TGN), as the primary hubs for sorting unconventional cargoes [21]. We therefore hypothesized that TGN‐anchored BioID2 would provide an effective complement to ER‐based profiling by capturing both canonical and unconventional secretory factors.

We first engineered two discrete constructs: ER‐BioID2 (KDEL‐tagged) and Golgi‐BioID2 (fused to the eNOS^1‐33^ peptide for TGN localization) [24] (Figure 1A). Initial validation in A549 cells confirmed high subcellular fidelity; high‐resolution confocal microscopy demonstrated that HA‐tagged enzymes and their corresponding biotinylated signals precisely co‐localized with organelle markers Calnexin (ER) and TGN46 (Golgi) (Figure 1B). Immunoblotting of cell lysates and culture supernatants revealed robust, biotin‐dependent labeling across both intracellular and extracellular fractions, establishing the TGN‐anchored system as a potent tool for secretome capture (Figure 1C).

**FIGURE 1:**
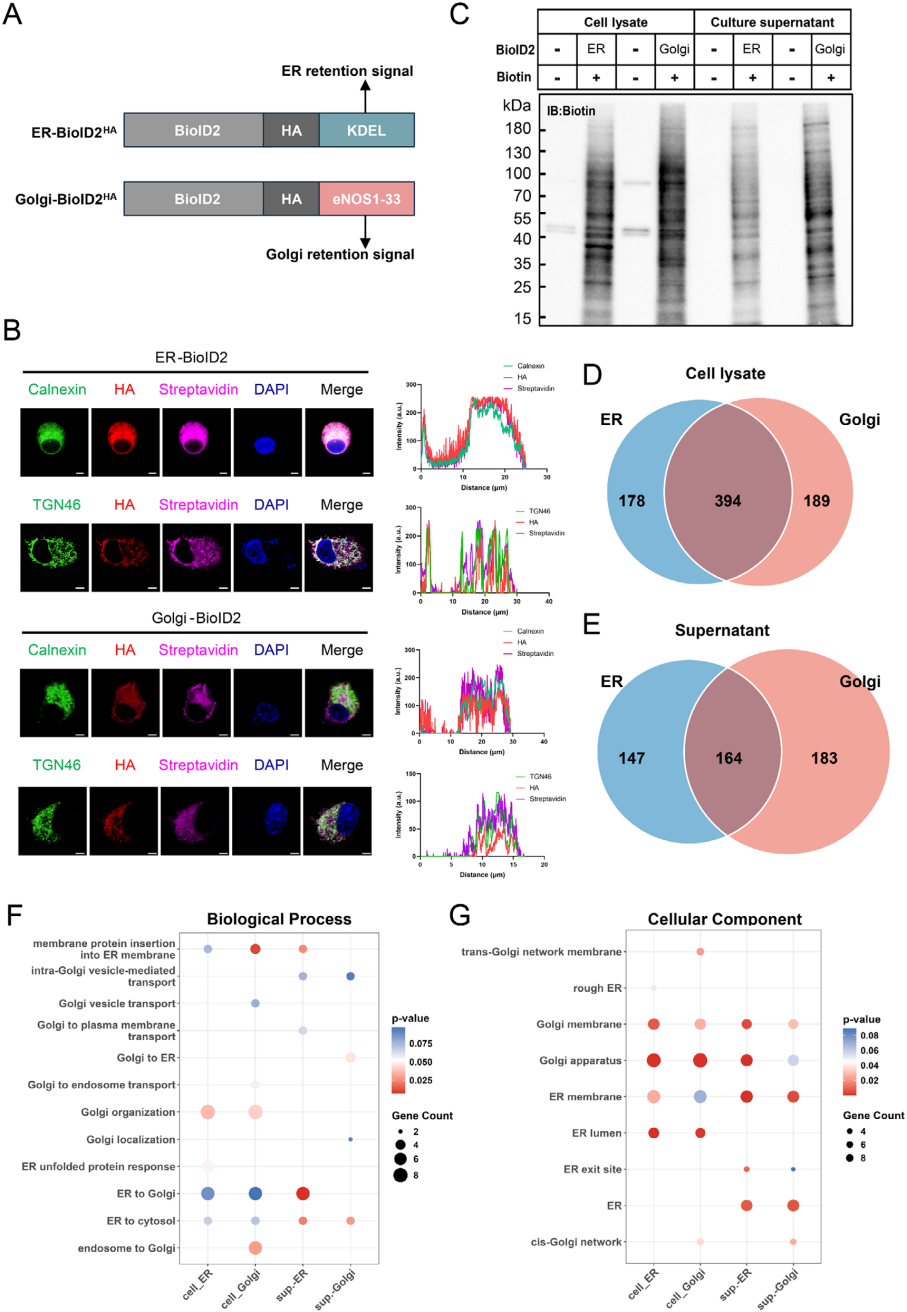
Proximity Labeling and Proteomic Profiling of the Secretory Pathway via ER‐ and Golgi‐anchored BioID2. (A) Schematic of ER‐BioID2 (KDEL‐tagged) and Golgi‐BioID2 (eNOS^1‐33^ signal peptide‐tagged) fusion constructs. Both include the biotinylating enzyme BioID2 and an HA epitope tag. The KDEL peptide directs ER retention, while the eNOS signal peptide targets the Golgi. (B) Subcellular localization and biotinylation activity of ER‐BioID2 and Golgi‐BioID2 fusion proteins in A549 cells. The up panel shows cells transfected with ER‐BioID2, where HA labeling indicates the localization of the BioID2 fusion protein in the endoplasmic reticulum (ER), with Calnexin marking the ER. Streptavidin labels biotinylated proteins. Additionally, cross‐labeling with the Golgi marker TGN46 reveals the spatial relationship between HA (BioID2) and streptavidin (biotinylated proteins). The down panel displays cells transfected with Golgi‐BioID2, where HA labeling indicates the localization of the BioID2 fusion protein within the Golgi apparatus, with TGN46 marking Golgi. Streptavidin again labels biotinylated proteins, and cross‐labeling with the ER marker Calnexin demonstrates the spatial relationship between HA (BioID2) and streptavidin (biotinylated proteins) in these cells. Line scan analysis showed the quantification of average nuclear intensity from immunofluorescence data for the indicated markers. Scale bar = 5 µm. (C) Biotinylation profiling of secretory proteins. Cell lysates and culture supernatants from BioID2 ‐expressing cells (±biotin treatment) were analyzed by streptavidin pull‐down and immunoblotting. Distinct biotinylation patterns were observed in. (D,E) Comparative proteomic landscape of the cellular and secreted biotinylated proteins. Venn diagrams illustrate the overlap and unique protein sets identified in (D) cell lysates and (E) culture supernatants (*n* = 2 biological replicates per group). (F,G) Functional enrichment of the BioID2‐captured proteome. Dot plots illustrate the top enriched (F) Biological Processes (BP) and (G) Cellular Components (CC) for proteins identified in both lysates and supernatants. The size of the dots represents the Gene Count associated with each term. The color gradient indicates the p‐value, where red dots denote significant enrichment (*p* < 0.05) and blue dots indicate non‐significant terms (*p* > 0.05). Confocal and gel imaging experiments were independently repeated at least twice with similar results.

To investigate how these strategies resolve the secretome, we conducted LC‐MS/MS analysis of biotinylated proteins in cell lysates and culture media. We identified 572 and 583 high‐confidence protein groups in the ER‐BioID2 and Golgi‐BioID2 cell lysates, respectively (Figure 1D and Table S1). In parallel, analysis of biotinylated proteins in the cell culture media—the extracellular secretome—yielded 311 (ER‐BioID2) and 347 (Golgi‐BioID2) proteins, respectively (Figure 1E and Table S2). While a shared core represents the fundamental secretory machinery, Gene Ontology (GO) revealed striking functional divergence of the unique proteins in ER‐ or Golgi‐initiated trafficking pathways. In general, ER‐unique proteins were significantly enriched in “ER unfolded protein response” and “ER to Golgi” pathways (Figure 1F), consistent with early‐stage quality control. In contrast, Golgi‐exclusive factors exhibited enrichment for “vesicular transport” (e.g., endosome‐to‐Golgi trafficking) (Figure 1F). Cellular Component (CC) analysis confirmed that the Golgi‐BioID2 proteome preferentially localized to the cis‐ and trans‐Golgi networks, underscoring its superior resolution for capturing post‐ER sorting events and unconventional cargoes (Figure 1G).

### 2.2 Synergistic Dual‐Anchoring Achieves Comprehensive Secretome Mapping

Building upon the distinct compartmental profiles, we developed the Dual‐Organelle Secretome Conjugation (DuO‐SC) platform to capture the full breadth of the secretome—encompassing both canonical and unconventional pathways. DuO‐SC utilizes a bicistronic E/G‐BioID2 construct, separated by an IRES element, to co‐express KDEL‐tagged and eNOS^1‐33^‐tagged BioID2 enzymes within the same cell. This strategy ensures that the secretory proteins bypassing the ER are biotinylated at the TGN sorting hub (Figure 2A). Immunofluorescence confirmed the high‐fidelity dual localization of the HA‐tagged BioID2 within both secretory hubs (Figure 2B). Moreover, Western blots revealed that while DuO‐SC maintained a cellular labeling profile comparable to its single‐compartment counterparts (Figure 2C), its performance in the culture supernatants was markedly superior, suggesting an enhanced capture of the extracellular proteome (Figure 2D). In addition, the bicistronic construct was designed to ensure coordinated expression of ER‐ and Golgi‐targeted BioID2 within the same cell. Compared with co‐transfection of separate ER‐BioID2 and Golgi‐BioID2 plasmids, the bicistronic vector exhibited enhanced biotinylation efficiency in both cellular and secreted protein fractions (Figure S1), supporting its use for comprehensive secretome labeling.

**FIGURE 2:**
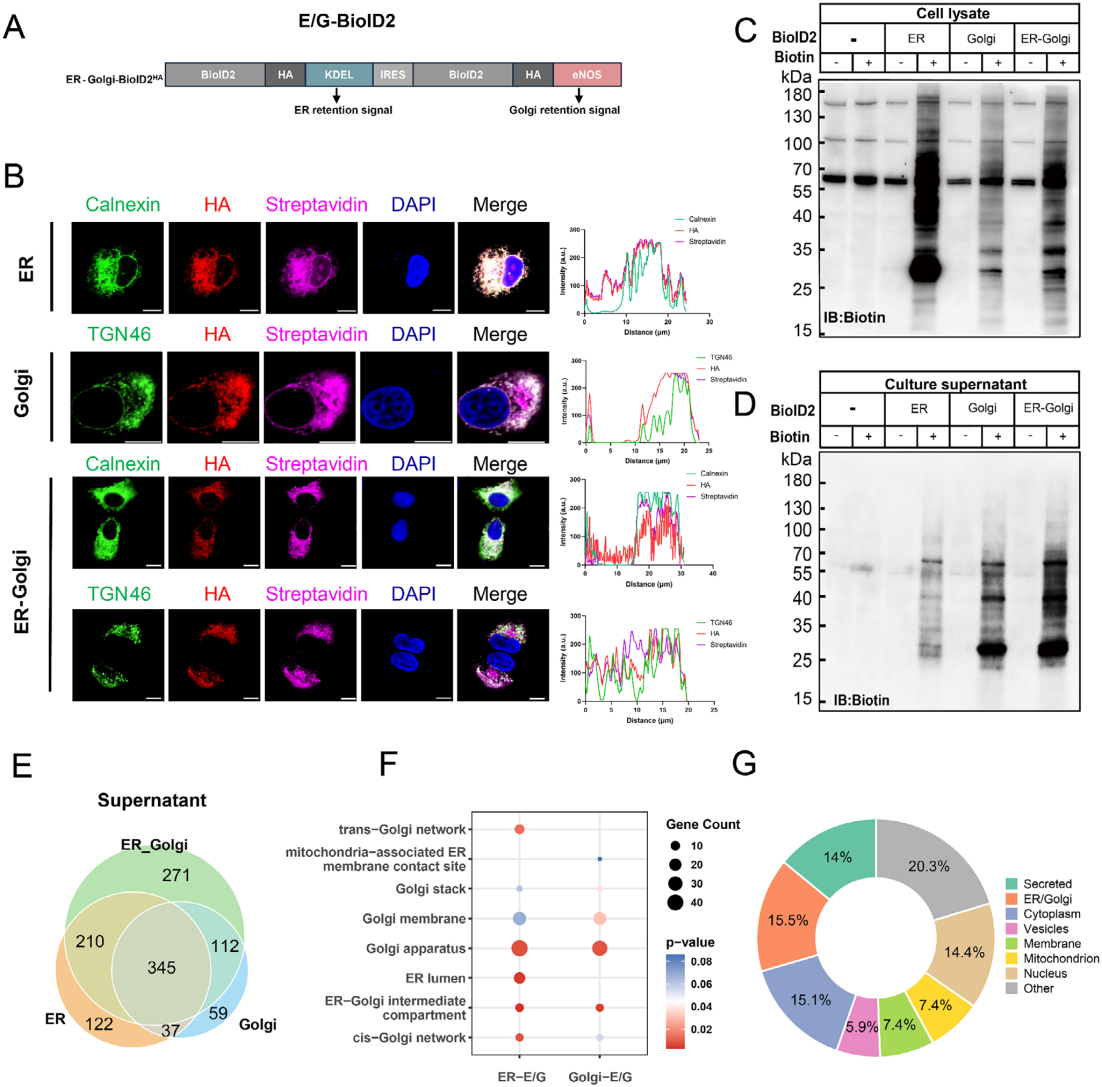
Characterization and Enrichment Analysis of the Secreted Proteome via the E/G‐BioID2 System. (A) Schematic illustration for secretory protein labeling by ER‐ anchored BioID2 and Golgi‐anchored BioID2. (B) Representative confocal images showing the subcellular localization of BioID2 (HA, red) and its biotinylation activity (streptavidin, pink) in A549 cells. Line scan analysis showed the quantification of average nuclear intensity from immunofluorescence data for the indicated markers. Scale bar = 10 µm. (C–D) Immunoblot analysis of biotinylated proteins in (C) cell lysates and (D) culture supernatants from A549 cells transfected with a control vector, ER‐BioID2 (ER), or Golgi‐BioID2 (Golgi). Results confirm efficient biotinylation and subsequent secretion of labeled proteins from both compartments; notably, the combined E/G‐BioID2 system achieved superior enrichment of biotinylated proteins in the culture supernatant compared to individual organelle targeting. (E) Comparative analysis of biotinylation efficiency in 293T cell supernatants. The results further validate that the integrated E/G‐BioID2 approach provides significantly broader labeling coverage of supernatant proteins than using either ER‐BioID2 or Golgi‐BioID2 alone (*n* = 2 biological replicates per group). (F) Cellular Component analysis of ER‐E/G shared proteins and Golgi‐E/G shared proteins in supernatant. The size of the dots represents the Gene Count associated with each term. The color gradient indicates the p‐value, where red dots denote significant enrichment (*p* < 0.05) and blue dots indicate non‐significant terms (*p* > 0.05). (G) Functional classification and subcellular distribution of the 271 E/G‐BioID2‐specific proteins. The analysis reveals a diverse proteomic landscape captured by the integrated system. Confocal and gel imaging experiments were independently repeated at least twice with similar results.

Quantitative evaluation via LC‐MS/MS identified a fundamental core of 345 proteins shared by the secretomes across all platforms (Figure 2E), representing the fundamental secretory framework. Intersectional analysis revealed that DuO‐SC effectively consolidated the labeling strengths of both organelle‐specific platforms. Specifically, a subset of 555 proteins was shared between the ER and DuO‐SC and was enriched for terms related to “ER lumen” (Figure 2F). In comparison, 457 proteins were shared between the Golgi and DuO‐SC conditions. Intriguingly, this Golgi‐ DuO‐SC overlapping proteome encompassed critical mitochondrial components, including TOMM70, TOMM22, and TOMM40. As core subunits of the Translocase of the Outer Mitochondrial Membrane (TOM) complex, these proteins are essential for mitochondrial protein translocation and serve as structural pillars of mitochondria [26]. Their robust enrichment could result from the presence of mitochondria‐associated membranes (MAMs), which form specialized physical contact sites between the ER and mitochondria. Because proximity‐labeling enzymes act within a finite labeling radius, proteins residing at ER‐mitochondrial contact interfaces may become accessible to labeling from the ER compartment. In addition, increasing evidence suggests that mitochondria‐derived vesicles and mitochondria‐associated trafficking pathways can communicate with components of the secretory system, potentially facilitating the recovery of mitochondrial proteins in extracellular fractions. Future investigations will address these hypotheses.

In addition, DuO‐SC identified 271 unique proteins entirely undetected by single‐compartment methods (Figure 2E,G). This represents a more than 120% increase in unique proteomic coverage compared to the conventional ER‐anchored system. Functional categorization of this unique dataset revealed pivotal elements of the vesicular machinery, including SEC61B, a critical component of the ER translocation apparatus, and STX6, a crucial SNARE protein mediating vesicular fusion. By bridging the secretory pathways that are previously “inaccessible”, DuO‐SC establishes a robust approach that is highly complementary to existing strategies for dissecting the secretome.

### 2.3 In Vivo Adipose Secretome Profiling Using the DuO‐SCOUT System

To enable comprehensive mapping of the adipose secretome in vivo, we generated a transgenic mouse line carrying a floxed E/G‐BioID2 cassette. Crossing this line with Adipoq‐Cre mice, we obtained the DuO‐SCOUT (Dual‐Organelle Secretome Conjugation and Organ‐Uptake Tracking) model with adipose‐specific co‐expression of KDEL‐tagged and eNOS^1‐33^‐tagged BioID2 enzymes (Figure 3A). Following one week of systemic biotin administration, immunofluorescence of HA‐tagged BioID2 confirmed precise co‐localization with biotinylated signals within the ER and Golgi apparatus of mature adipocytes (Figure 3B). Western blot analysis further revealed robust protein biotinylation across adipose depots, with epididymal white adipose tissue (eWAT) exhibiting higher biotinylation levels than subcutaneous depots (sWAT), likely reflecting depot‐specific metabolic kinetics (Figure 3C).

**FIGURE 3:**
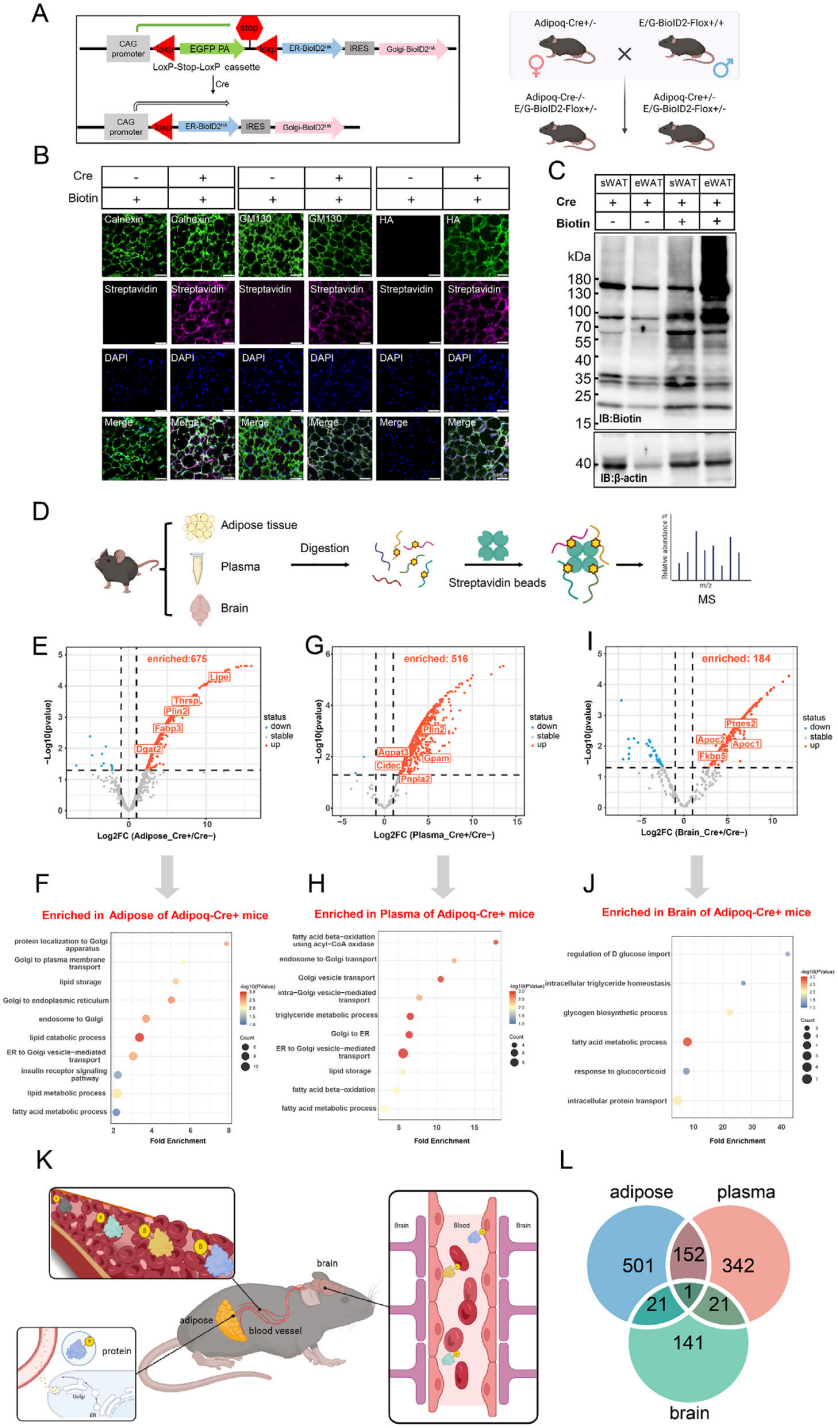
Adipose‐Specific Activation of the E/G‐BioID2 System for In Vivo Mapping of the Adipose Secretome. (A) Cre‐mediated recombination induces expression of E/G BioID2 from the flox Allele. The left panel presents a schematic diagram of the gene structure. The right panel displays transgenic mice (DuO‐SCOUT mice) generated by crossing Adipoq‐cre^+/−^ mice with E/G‐BioID2^flox/flox^ mice, resulting in adipose tissue‐specific expression of E/G‐BioID2. (B) Immunofluorescence analysis of adipose tissue in DuO‐SCOUT mice. ER and Golgi apparatus were marked using Calnexin and GM130, respectively. BioID2 fusion proteins were detected with anti‐HA, and biotin‐labeled proteins were visualized using streptavidin (pink). Streptavidin staining confirms robust in situ biotinylation of proteins within the adipocyte secretory compartments. Scale bar = 50 µm. (C) Immunoblot analysis of sWAT and eWAT lysates from DuO‐SCOUT mice. The results demonstrate that the enrichment of the biotinylated proteome is strictly dependent on the administration of exogenous biotin, showing low background labeling in non‐injected DuO‐SCOUT controls. This confirms the high signal‐to‐noise ratio and substrate‐controllability of the E/G‐BioID2 system in different adipose depots in vivo. (D) Schematic workflow for the extraction of adipose tissues, plasma, and brain tissues, followed by MS‐based analysis. (E,G,I) Volcano plots highlighting differentially enriched biotinylated proteins in (E) adipose tissue, (G) plasma, and (I) brain of Adipoq‐Cre+ mice versus control mice (*n* = 2 biological replicates per group). Red symbols denote significantly enriched proteins (*p* ≤ 0.05 and fold‐change ≥2), with key adipose‐derived factors highlighted. Blue symbols represent endogenously biotinylated proteins enriched in control samples. (F,H,J) Gene Ontology (GO) enrichment analysis revealing the functional landscape of the biotinylated proteome in (F) adipose, (H) plasma, and (J) brain of Adipoq‐Cre+ mice. (K) Schematic illustration of the proposed adipose–plasma–brain communication axis, where proteins secreted from adipose tissue enter systemic circulation and reach the brain to potentially modulate central functions. (L) Venn diagram illustrates the overlap of significantly enriched proteins across the three compartments (adipose, plasma, and brain), identifying candidate inter‐organ communicators (*n* = 2 biological replicates per group). Serpina6 was identified as the sole candidate enriched in adipose ER/Golgi hubs, plasma, and brain, suggesting its active secretion from adipocytes and subsequent trafficking to the central nervous system. Confocal and gel imaging experiments were independently repeated at least twice with similar results.

Adipokines are adipose‐derived factors that enter circulation and can cross the blood‐brain barrier to influence central processes such as energy balance and neuroinflammation [3]. To investigate the adipose‐brain axis, we performed LC‐MS/MS analysis of biotinylated proteins across three key compartments: adipose (source), plasma (transport), and brain (target) (Figure 3D). We identified 675, 516, and 184 proteins significantly enriched (≥2‐fold, *p* ≤ 0.05, Figure 3E,G,I and Tables S3–S5) in the cre^+^ group in comparison to the cre^−^ mice, respectively. GO term enrichment confirmed that these proteomes were strongly associated with ER‐Golgi trafficking and fatty acid metabolism, supporting their secretory nature and adipose origin (Figure 3F,H,J).

Intersectional analysis of these upregulated proteomes provided a high‐resolution map of systemic transit (Figure 3L). We identified 153 proteins shared between adipose and plasma, including Igf1, Lbp, and Pcsk9, which are recognized adipokines with a signal peptide [27, 28, 29]. This confirms that DuO‐SCOUT effectively captures factors released via classical pathways and tracks them into the circulation. Furthermore, 22 proteins overlapped between plasma and brain, including the appetite regulator Dbi, a novel adipokine that plays a significant role in appetite regulation, energy homeostasis, and obesity [30]. Notably, Serpina6 (also known as corticosteroid‐binding globulin) was enriched across all three compartments. Given that adipose tissue is a known source of Serpina6 and participates in systemic cortisol regulation [31, 32, 33], its detection across the entire axis validates DuO‐SCOUT as a powerful tool for tracing the “traveling proteome” from peripheral synthesis to central uptake. The functional role of Serpina6 in the brain remains to be investigated.

### 2.4 Tracking the Remodeling of Adipose Secretome in Diet‐Induced Obesity

To investigate how metabolic stress shapes the adipose secretome, we established a diet‐induced obesity (DIO) model in which DuO‐SCOUT mice on a high‐fat diet (HFD) reached a peak body mass of ∼55g by week 20 (Figure 4A and Figure S3). Following systemic biotin labeling, enrichment, and LC‐MS/MS analysis, we identified 29 plasma proteins with significant abundance changes (≥ 2‐fold change and *p* ≤ 0.05). Notably, 11 factors were significantly upregulated in HFD mice, including the adipose‐derived factors SERPING1 and APOA2 (Figure 4B). Functional GO enrichment of these upregulated factors identified terms such as “inflammatory response” and “cellular response to cytokine stimulus” (Figure 4C). Specifically, the elevation of SERPING1, a known regulator of the complement system, suggests that obese adipose tissue acts as a systemic “inflammatory outpost,” actively broadcasting signals that drive chronic low‐grade inflammation.

**FIGURE 4:**
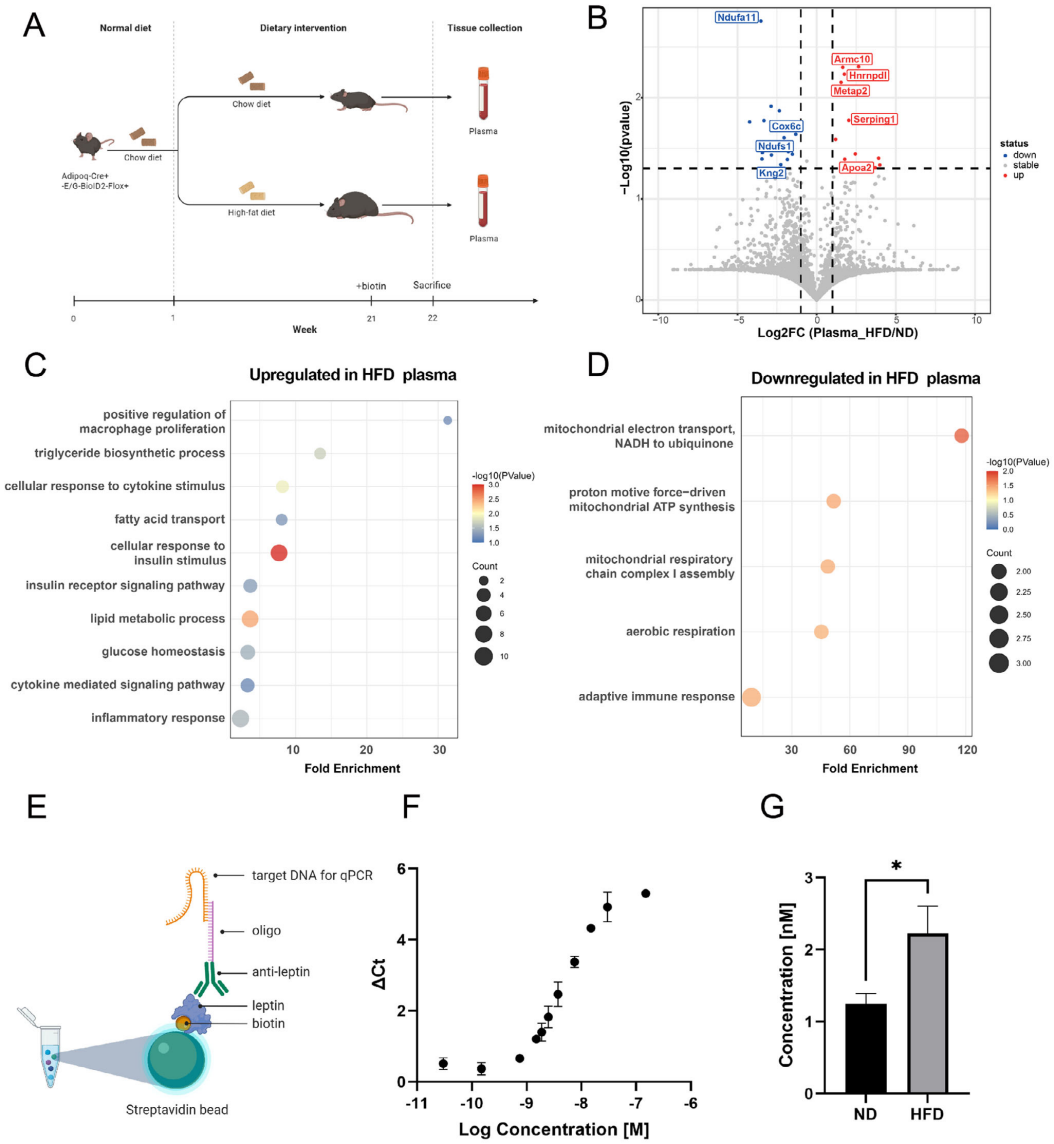
Characterization of the Adipose‐Derived Secretome in Obese Mouse Models. (A) Schematic of the experimental timeline for High‐Fat Diet (HFD) and Normal Diet (ND) feeding, followed by plasma collection. (B) Differentially expressed adipose‐derived proteins in plasma. Quantitative LC‐MS/MS identified 29 proteins with significant abundance shifts (≥ 2‐fold change and *p* ≤ 0.05) between HFD and ND groups (*n* = 2 biological replicates per group). HFD‐upregulated factors (e.g., SERPING1 and APOA2), indicate a shift toward a pro‐inflammatory secretory profile, reflecting the role of obese adipose tissue as a systemic inflammatory mediator. In contrast, proteins downregulated in the HFD group (e.g., NDUFA11, COX6C) were predominantly associated with oxidative phosphorylation and thermogenesis, suggesting a suppression of mitochondrial bioenergetics. (C,D) Divergent functional signatures of the adipose‐derived plasma proteome in HFD versus ND mice. (C) Biological Process (BP) enrichment analysis of significantly upregulated proteins in the HFD plasma, revealing a predominant shift toward inflammatory responses. (D) BP analysis of proteins downregulated in the HFD group (enriched in the ND group), highlighting a significant loss of mitochondrial ATP synthesis pathways. This reciprocal shift—characterized by a decline in fat‐burning efficiency and a concomitant rise in systemic inflammation—likely exacerbates the metabolic dysregulation observed in diet‐induced obesity. (E) Diagram of detecting biotinylated leptin in blood. (F) Biotinylated leptin standard curve. Error bars, standard deviation. *n* = 4 replicates per condition. (G) Comparison of biotinylated leptin level between ND and HFD group in plasma. Error bars, standard deviation. *n* = 4 mice per condition. *t*‐test, ^*^p <0.05.

While LC‐MS successfully identified inflammatory signatures, it did not detect leptin, a key adipokine that coordinates the adipose‐brain axis in regulating appetite and metabolism [34, 35, 36]. In obesity, elevated leptin levels are a primary driver of systemic low‐grade inflammation and metabolic dysfunction [37, 38]. Due to its small size (∼16 kDa), low stoichiometry in circulation, and rapid clearance, it is difficult to capture leptin dynamics using quantitative LC‐MS. To bridge this gap, we integrated the DuO‐SCOUT platform with a highly sensitive, antibody‐based protein‐to‐DNA detection assay (Figure 4E). This method captures de novo biotinylated leptin from adipose tissue using streptavidin‐coated beads and utilizes an anti‐leptin antibody conjugated to a unique oligonucleotide to translate the protein signal into a DNA signal for quantitative PCR. This approach demonstrated a detection sensitivity of 0.9 nM (Figure 4F). Using this refined detection limit, we observed a significant increase in adipose‐derived leptin flux in HFD mice compared to normal diet (ND) controls (Figure 4G), in line with previous findings that obesity leads to a heightened adipose‐derived leptin flux [37]. These results indicate that the DuO‐SCOUT mice can integrate with protein‐to‐DNA detection technologies to track adipokine changes that are invisible to standard mass spectrometry.

### 2.5 Spatial Delineation of the Adipose‐Brain Axis of Leptin Signaling

To resolve the precise anatomical distribution of adipose‐derived leptin within the brain, we developed a Biotin‐Specific Proximity Amplification (BSPA) assay (Figure 5A). BSPA utilizes a dual‐probe recognition system to selectively detect biotinylated proteins with high spatial fidelity. The BSPA framework comprises: (i) an anti‐leptin antibody conjugated with a unique oligonucleotide (Probe A); (ii) streptavidin conjugated with a second unique oligonucleotide (Probe B); and (iii) a connector oligonucleotide that facilitates circular DNA template formation only when all components are in extreme proximity (< 40 nm). This template undergoes rolling circle amplification (RCA), generating a concentrated DNA “nanoball” detectable via fluorescent probes. Signals are produced exclusively when the biotin tag is present on leptin, ensuring high specificity (Figure 5B). When applied to adipose sections, BSPA showed markedly elevated signals of biotinylated leptin in HFD adipose tissue compared to ND controls (Figure 5C,D). No signal was detected in samples without biotin labeling, confirming the high specificity of BSPA.

**FIGURE 5:**
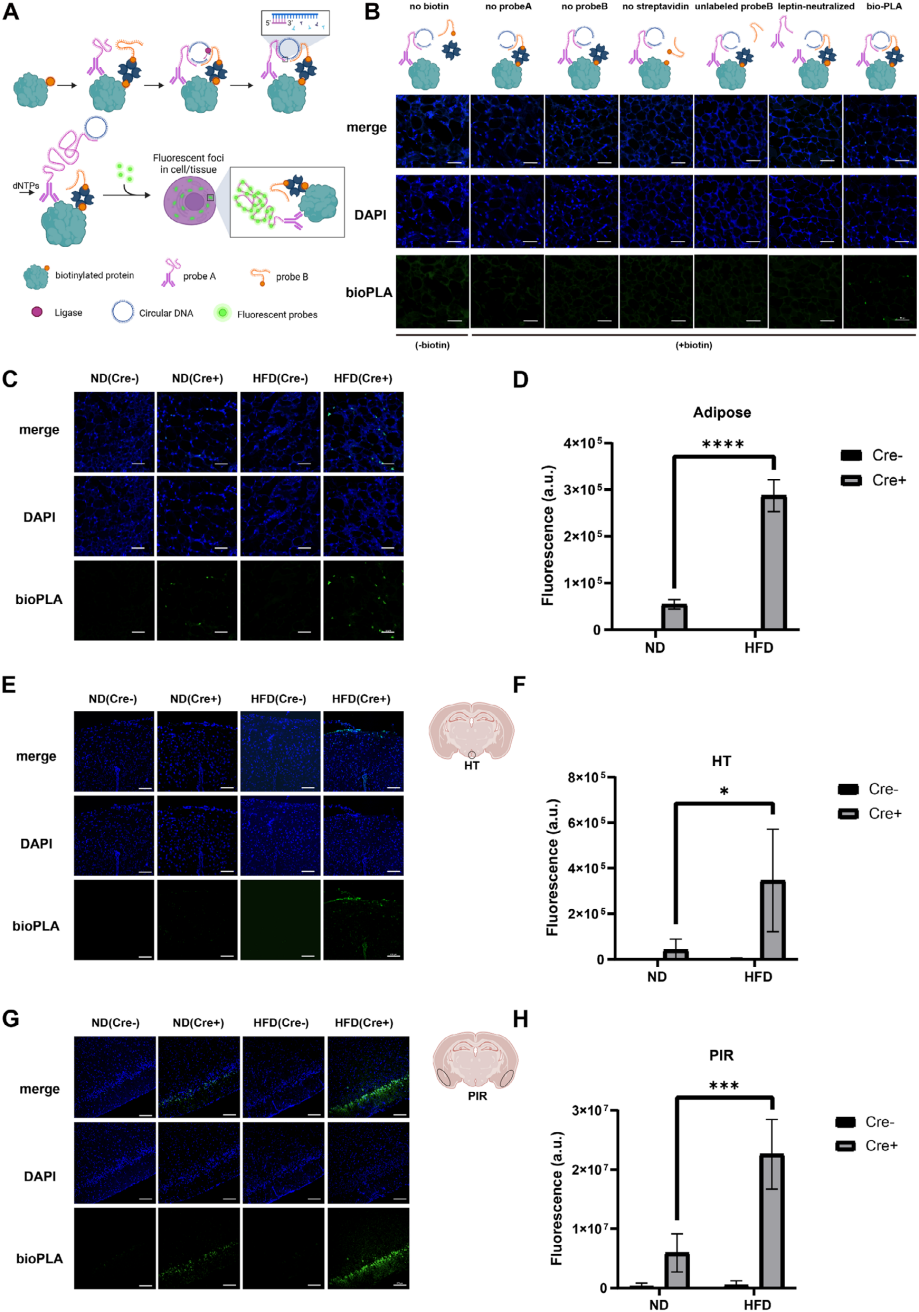
In Situ Visualization of Adipose‐Derived Proteins via BSPA in Adipose and Brain Tissues. (A) Principles of BSPA for biotinylated proteins detection. The system utilizes a dual‐probe recognition mechanism involving a target‐specific antibody (Probe A) and a biotin‐targeting probe (Probe B). Upon binding in close proximity (<40 nm), connector oligonucleotides mediate the formation of a circular DNA template for rolling circle amplification (RCA), generating a localized fluorescence signal (DNA “nanoball”) that represents the tissue‐specific protein of interest. (B) BSPA for in situ imaging of bio‐leptin in adipose tissue from transgenic mice. Representative fluorescence images of bio‐leptin visualized by BSPA in positive adipose tissues. The bright green dots indicate strong signal of bio‐leptin, and cell nuclei are shown in blue (DAPI). (C–D) Adipose tissue: Representative BSPA imaging (C) and quantification (D) of biotinylated leptin in ND and HFD mice using FIJI. The HFD group exhibits a dramatic increase in local bio‐Leptin signals. Error bars, standard deviation. *n* = 4 mice per condition. *t*‐test, ^****^p <0.0001. (E–H) Brain regions: Comparative BSPA mapping of adipose‐derived leptin in the Hypothalamus (HT; E–F) and Piriform Cortex (PIR; G–H). The consistent elevation of BSPA signals in these regions under HFD conditions confirms the enhanced systemic trafficking of adipokines to the brain. Error bars, standard deviation. *n* = 5 mice per condition. *t*‐test, ^*^p <0.05, ^***^p <0.001.

Next, we applied BSPA to brain sections of DuO‐SCOUT mice following ND and HFD to investigate the spatial distribution of adipose‐derived leptin. Fluorescent signals of biotinylated leptin were predominantly localized to the hypothalamus (HT), with signal intensity in HFD mice approximately eightfold higher than in ND controls (Figure 5E,F). This aligns with the function of the hypothalamus in integrating peripheral hormonal cues to regulate metabolism [39, 40]. Moreover, BSPA also revealed significant accumulation of biotinylated leptin in the piriform cortex (PIR)—a limbic structure associated with olfactory processing and emotional regulation—with HFD mice showing approximately fourfold stronger fluorescence signals than ND controls (Figure 5G,H). While previous studies reported the presence of leptin in the PIR [41], our data provide the first visual evidence of heightened adipose‐derived leptin flux to this region during obesity, suggesting a direct link between the peripheral secretome and alterations in sensory or neurobehavioral responses. No significant difference in leptin BSPA signals was observed between the ND and HFD group in other brain regions (Figure S5).

It is important to validate that the BSPA signals are in the brain parenchyma rather than from residual blood contaminations. To address this, we performed immunofluorescence staining of CD31, a well‐established endothelial marker, to delineate the cerebral vasculature. As shown in Figure S7, the majority of CD31‐positive blood vessels exhibited little or no BSPA signal, indicating effective vascular clearance following tissue perfusion. Furthermore, in regions exhibiting strong BSPA signals, including the PIR and HT, the biotinylated signals displayed minimal colocalization with CD31‐positive vascular structures. These results demonstrate BSPA as a robust platform for resolving the distribution of adipose‐derived leptin in the brain parenchyma.

### 2.6 Leptin Triggers a Neuroinflammatory Signature

To determine the functional consequences of the heightened adipose‐to‐brain signaling axis, we performed laser capture microdissection (LCM) followed by RNA‐seq analysis of the HT and PIR regions from HFD and ND mice (Figure 6A). In the HT region of HFD, we identified 913 upregulated and 340 downregulated genes (≥ 2‐fold change, *p* ≤ 0.05; Figure 6B and Figure S9A). STRING network analysis (Figure 6C) identified pro‐inflammatory factors as the core upregulated signatures, including Cxcl9, Nos2, Slc30a8, and Fosl1, which mediate immune cell recruitment, cytokine signaling, cellular homeostatic disruption and inflammatory amplification. In contrast, downregulated genes (e.g., Ppara, Fth1) are anti‐inflammatory regulators that suppress inflammatory cascades and protect against oxidative damage. GO analysis of upregulated genes in the HT region revealed enrichment in inflammation‐related pathways, including cellular response to interferons and positive regulation of TNF production (Figure 6D). These findings indicate that obesity induces robust activation of inflammatory signaling in the HT, positioning inflammation as a key driver of hypothalamic dysfunction in obese mice.

**FIGURE 6:**
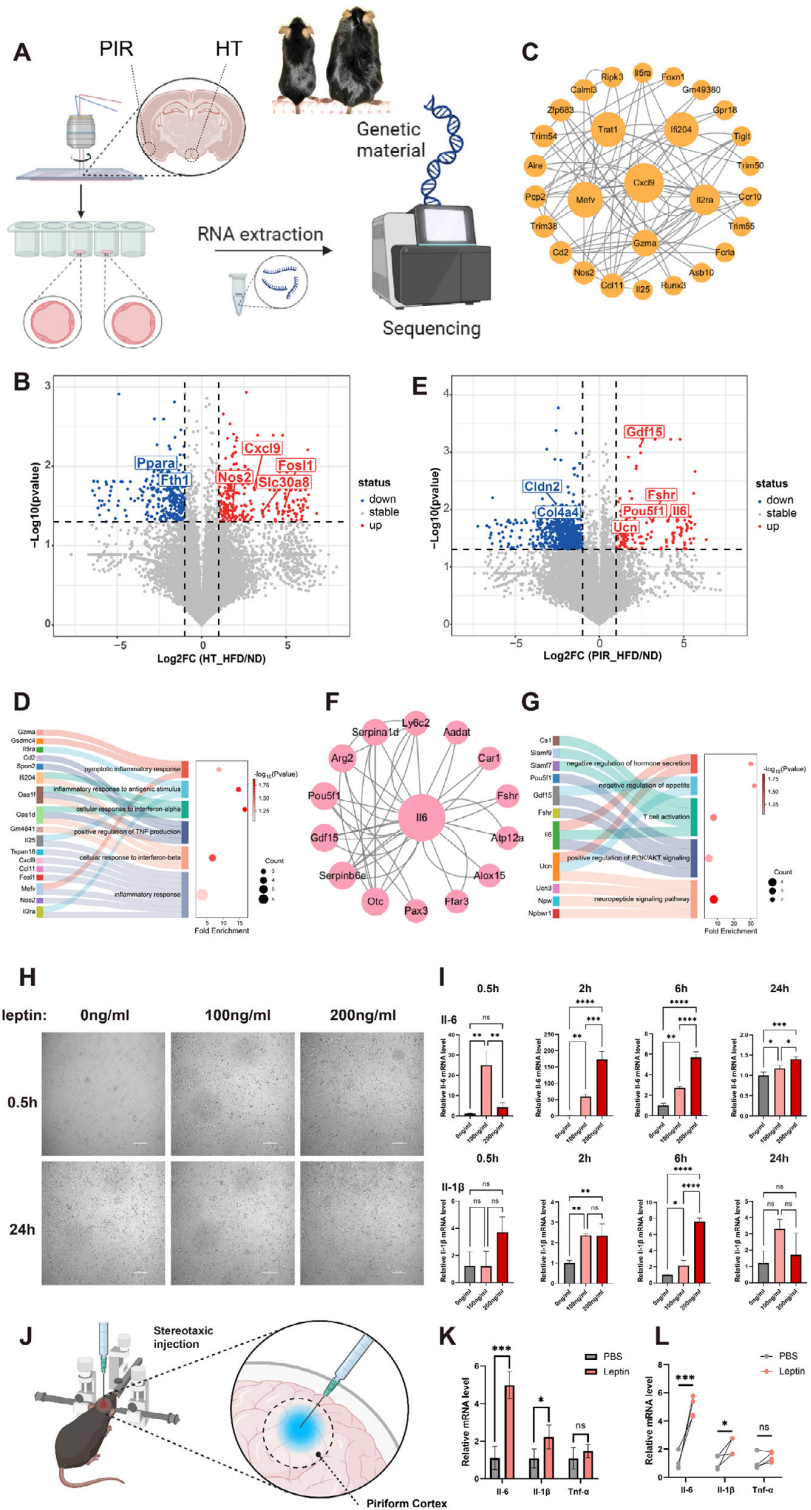
Spatially Resolved Transcriptomic Profiling of Obesity‐Induced Brain Inflammation and Metabolic Dysregulation. (A) Schematic of the LCM‐seq workflow used to isolate and analyze RNA from the Hypothalamus (HT) and Piriform Cortex (PIR) of ND and HFD mice. (B) Volcano plot of differentially expressed genes (DEGs) in the HT. HFD mice exhibit a significant pro‐inflammatory shift, characterized by the upregulation of inflammatory mediators (e.g., Cxcl9, Nos2, Slc30a8, Fosl1; red) and the downregulation of essential metabolic and anti‐inflammatory markers (e.g., Ppara, Fth1; blue) (*n* = 3 biological replicates per group). This gene expression pattern reflects a compromised neuroprotective environment in the obese hypothalamus. (C) STRING functional protein‐protein interaction (PPI) network highlighting a coordinated pro‐inflammatory cluster centered on Cxcl9. (D) Sankey dot plot of GO enrichment terms for HFD‐upregulated genes in HT, revealing a predominant shift toward interferon signaling (alpha/beta), TNF production, and inflammatory responses. (E) Volcano plot of DEGs in the PIR. Notable HFD‐upregulated genes include pro‐inflammatory and metabolic modulators (e.g., IL6, GDF15, Pou5f1, Ucn and Fshr) (*n* = 3 biological replicates per group). Significantly, the downregulation of tight junction (Cldn2) and basement membrane (Col4a4) components suggests a potential compromise in blood‐brain barrier (BBB) integrity. (F) STRING network analysis identifies Il6 as a central hub for PIR‐specific inflammatory signaling. (G) Sankey dot plot of GO terms for HFD‐upregulated genes in the PIR. The enrichment profile reveals a specialized response to obesity‐induced metabolic stress within the Piriform Cortex. The activation of T cell mediated immunity indicates a localized neuro‐inflammatory environment, potentially triggered by the sustained influx of adipose‐derived signals (e.g., Leptin). Simultaneously, the enrichment in negative regulation of appetite and PI3K/AKT signaling suggests that the PIR functions as an extra‐hypothalamic site for metabolic feedback. This molecular signature captures the brain's coordinated attempt to suppress appetite and modulate energy metabolism through the PI3K/AKT cascade, responding directly to increased peripheral adipokine signals. (H) Representative phase‐contrast images of primary cortical cells. Leptin treatment (up to 24 h) induced no overt cytotoxicity or morphological alterations, confirming the observed molecular changes are regulatory rather than degenerative. (I) Quantification of canonical inflammatory markers, Il6 and Il1β, mRNA expression in primary cortical cells treated with varying concentrations of leptin for different durations. Gene expression levels were measured by qPCR and presented as bar graphs. Data are presented as mean ± SD. Each condition was measured using three independent primary cortical cell cultures (*n* = 3). *t*‐test, ns = not significant, ^*^p < 0.05, ^**^p <0.01, ^***^p <0.001, ^****^p <0.0001. (J) Schematic illustration of the stereotaxic injection of recombinant leptin into the piriform cortex. (K) Bar‐graph quantification of canonical inflammatory markers, Il6, Il1β and TNF‐α after extracting RNA. Data are presented as mean ± SD (*n* = 4). *t*‐test, ns = not significant, ^*^p < 0.05, ^***^p <0.001. (L) Paired‐line quantification of canonical inflammatory markers, Il6, Il1β and TNF‐α after extracting RNA. Data are presented as mean ± SD (*n* = 4). *t*‐test, ns = not significant, ^*^p < 0.05, ^***^p <0.001.

In the PIR region, we identified 436 upregulated and 938 downregulated genes in HFD mice (≥ 2‐fold change, *p* ≤ 0.05; Figure 6E and Figure S9B). Notably, Il6, which drives both acute and chronic inflammatory responses, was significantly upregulated in the HFD group (Figure 6F). Other upregulated genes (e.g., Gdf15, Pou5f1, Ucn, Fshr) are also associated with inflammatory and stress signaling and insulin resistance, contributing to immune activation, vascular/cellular remodeling, and metabolic dysregulation. Interestingly, several downregulated genes (e.g., Cldn2, Col4a4) encode structural components of the blood‐brain barrier. Their reduced expression is consistent with molecular remodeling of the neurovascular microenvironment under metabolic stress in obesity. GO analysis revealed enrichment of leptin‐associated pathways, including positive regulation of PI3K/AKT signal transduction (Figure 6G) [42]. In addition, leptin stimulation of primary cortical cells cultured in vitro exhibited a stark enhancement of Il6 and Il1β expression (Figure 6I), while no overt cytotoxicity or morphological alterations were observed (Figure 6H).

To investigate its pro‐inflammatory potential in vivo, we performed stereotaxic injection of leptin into PIR of one hemisphere. PBS was injected into the contralateral hemisphere of the same animal as an internal control. LCM analysis of inflammatory gene expression demonstrated that local leptin administration significantly increased the expression of Il6 and Il1β, but not Tnf‐α, in the injected PIR region (Figure 6J–L). These results provide direct in vivo evidence that elevated local leptin levels are sufficient to induce inflammatory signaling within the PIR. Collectively, these findings suggest that the adipose‐derived leptin flux captured by DuO‐SCOUT drives inflammatory activation and compromises structural integrity within the PIR, revealing it as a critical extra‐hypothalamic target of metabolic dysfunction.

## 3 Discussion

In this study, we present DuO‐SCOUT, which facilitates comprehensive labeling and profiling of the secretome in vitro and in vivo. When applied in conjunction with BSPA, we could track biotinylated adipokines in circulation and within recipient tissues with exquisite sensitivity. We propose DuO‐SCOUT as a robust framework for dissecting the secreted proteome in organ‐organ communications.

Recent advances in proximity‐dependent biotinylation have substantially expanded the ability to profile secretomes in vitro and in vivo. ER‐anchored BioID‐labeling strategies have proven highly effective for capturing proteins entering the classical secretory pathway. However, because these approaches rely primarily on labeling within the ER lumen, they are intrinsically biased toward proteins that engage the conventional ER‐to‐Golgi trafficking route and may underrepresent proteins utilizing post‐ER sorting mechanisms or unconventional secretion pathways. In contrast, DuO‐SCOUT employs simultaneous labeling at both the ER and Golgi compartments, thereby extending proteomic coverage across multiple stages of secretory maturation and increasing the probability of capturing proteins that may be incompletely represented by single‐organelle strategies. DuO‐SCOUT achieved a synergistic enhancement in biotinylation efficiency—identifying 120% more secreted proteins compared to the traditional ER single‐compartment labeling (Figure 2E). This unprecedented depth is instrumental for capturing the full complexity of the adipose secretome, particularly for adipokines that possess no classical signaling peptides for secretion [20]. The TGN‐anchored BioID could also potentially label proteins released by extracellular vesicles, as well as mitochondrial components (Figure 2G). The labeling specificity and efficiency of such proteins warrant further investigations.

Among currently available in vivo secretome‐labeling platforms, iSLET and related tissue‐specific biotinylation systems have provided important frameworks for tracking tissue‐derived proteins in circulation [16]. Nevertheless, the primary output of these approaches is generally the circulating secretome itself, whereas determining the ultimate tissue destination of secreted proteins remains challenging because of extensive dilution in blood and limited sensitivity in recipient organs. DuO‐SCOUT was designed to address this gap by coupling secretome labeling with BSPA organ‐uptake analysis. BSPA leverages the biotin tag introduced during DuO‐SC labeling and converts this molecular signature into an amplified DNA signal, enabling highly sensitive visualization of tissue‐derived proteins within recipient organs. Consequently, this enables interrogation of the entire trajectory from tissue of origin, to circulation, and ultimately to recipient tissues.

To substantiate in vivo applications, we established adipose‐specific DuO‐SCOUT mice to examine the adipose secretome (Figure 3). Comparative profiling of HFD and ND mice identified 29 differentially expressed proteins, including key factors closely linked to inflammatory cascades in the circulation (Figure 4B). Traditional methods for in vivo tracking of adipokines often face the challenge of massive systemic dilution and the selective nature of the blood‐brain barrier [43]. To address this, we developed BSPA that showed sub‐nanomolar sensitivity in detecting biotinylated leptin (Figure 4E,F). This revealed enhancements of leptin accumulation in circulation, HT, and PIR of HFD mice (Figures 4G and 5). We propose that the DuO‐SCOUT/BSPA integration will facilitate delineating the biology of additional adipokines in the near future.

Beyond its technical innovation to map the adipose secretome, our work provides a powerful toolkit for dissecting how peripheral metabolic signals interact with the central nervous system. By identifying the PIR—a critical hub for associative olfactory processing [44] —as a major target of leptin signaling in HFD mice (Figure 6), we expand the scope of obesity‐induced neuroinflammation beyond classical homeostatic centers. Future studies incorporating leptin receptor manipulation and cell‐type‐specific analyses, as well as genetic knockout models and behavioral studies, will provide further validation for the functional mechanisms of leptin in PIR.

Despite the robustness and sensitivity of DuO‐SCOUT, several limitations remain for future refinement. The first limitation arises from endogenous biotinylated proteins, particularly biotin‐dependent carboxylases, which generate background signals in metabolically active tissues. Although differential enrichment analysis minimizes their impact on secretome identification, extremely low‐abundance proteins may remain challenging to detect. Second, the current DuO‐SCOUT does not allow deconvolution of the relative contributions of individual adipose depots to the circulating or brain‐associated biotinylated proteome. The biotinylated proteins detected in plasma and brain represent the integrated output of the adipose compartment rather than a single depot. Third, while BSPA provides high‐resolution spatial mapping of individual proteins, it is restricted by the availability of antibodies and the number of simultaneous targets. Overall, we propose DuO‐SCOUT not as a replacement for existing secretome technologies, but rather as a highly complementary platform that integrates comprehensive secretome labeling with spatially resolved recipient‐organ tracking. The integration of DuO‐SCOUT with the emerging spatial proteogenomics will be essential to achieve a holistic map of the inter‐organ secretome that orchestrates systemic health and disease.

## 4 Experimental Section

### 4.1 Animal Models and Ethics Statement

All animal procedures were conducted in accordance with the animal care guidelines and regulations approved by the Department of Health of Hong Kong. The Adipoq‐Cre transgenic mouse line (B6.FVB‐Tg (Adipoq‐cre)1Evdr/J; Stock No: 028020; RRID: IMSR_JAX:028020) was purchased from The Jackson Laboratory. This strain expresses Cre recombinase specifically in mature adipocytes under the control of the mouse adiponectin (Adipoq) promoter/enhancer. Mice were maintained in a temperature‐controlled environment with a 12‐h light/dark cycle and provided ad libitum access to water and a standard chow or high‐fat diet (HFD, 60% kcal from fat) as indicated.

### 4.2 Primary Cortical Neuron Culture

Primary cortical neurons were isolated from C57BL/6J mouse embryos at embryonic day 18 (E18) as previously described [45]. Briefly, cerebral cortices were microdissected under a stereomicroscope, ensuring the removal of the meninges and the exclusion of the hippocampal formation. The isolated tissues were enzymatically dissociated and mechanically triturated to obtain a single‐cell suspension. Cells were seeded at a high density of 3 × 10^6^ cells per dish onto 60‐mm culture dishes coated with 0.1 mg/mL poly‐D‐lysine (Sigma, P0899). Neurons were maintained in Neurobasal medium supplemented with 2% (v/v) B27 and 0.25% (v/v) L‐glutamine (Invitrogen). To suppress the growth of overproliferating glial cells and ensure a highly purified neuronal culture, 12.5 mm L‐glutamic acid (Sigma, G8415) was added to the medium on day 5 in vitro (DIV 5). The neurons were cultured for a total of 10 days (DIV 10) to reach functional maturation, with partial medium exchange every 3 days. Mature cultures were subsequently utilized for in vitro leptin stimulation and downstream assays. Following treatments, cells were lysed, and total RNA was extracted and reverse‐transcribed into cDNA. For RT‐qPCR validation, the expression level of target genes was normalized to that of β‐actin (Actb), and the relative fold change in mRNA expression was calculated using the 2^−ΔΔCt^ method.

### 4.3 Cell Culture

Human embryonic kidney (HEK) 293T cells and the non‐small cell lung cancer (NSCLC) A549 cell line were purchased from the American Type Culture Collection (ATCC). Cells were maintained in Dulbecco's Modified Eagle Medium (DMEM) supplemented with 10% (v/v) fetal bovine serum (FBS) and 1% penicillin‐streptomycin. Cultures were incubated at 37°C in a humidified atmosphere containing 5% CO_2_.

### 4.4 Chemical and Enzymes

The DNA sequences were purchased from Shanghai Sangon Biological Engineering Technology & Services Co., Ltd. (Shanghai, China). The DNA sequences modified with Alexa488 or biotin were purchased from Shanghai Sangon Biological Engineering Technology & Services Co., Ltd. (Shanghai, China), and were purified by HPLC. T4 polynucleotide kinase, T4 DNA ligase and Phi29 DNA polymerase were purchased from Life Technologies (Carlsbad, CA). Oligonucleotide Conjugation Kit (ab218260) was purchased from abcam. Phusion High‐Fidelity DNA Polymerase and Phusion Hot Start Flex DNA Polymerase were purchased from Thermo. Agilent Protein 230 Kit was purchased from Tinhangtech. Recombinant Mouse Leptin Protein, CF, was purchased from R&D Systems. PET frameslide for laser microdissection was purchased from Leica. Color changing silica gel desiccant, liquid blocker super PAP pen, and cresyl violet were purchased from Beyotime.

### 4.5 Dietary Intervention

All animal procedures were approved by the Department of Health of Hong Kong. Adipoq‐Cre mice (B6.FVB‐Tg(Adipoq‐cre)1Evdr/J; Stock No: 028020) were purchased from The Jackson Laboratory. To generate the conditional E/G‐BioID2 overexpression line, the E/G‐BioID2 fragment was cloned downstream of a loxP‐Stop‐loxP (LSL) cassette under a pCAG promoter. The transgene was introduced into C57BL/6J fertilized eggs via pronuclear microinjection. Transgenic founders were crossed with Adipoq‐Cre mice to obtain adipose‐specific E/G‐BioID2‐expressing offspring, which were identified via PCR genotyping. Mice (6–8 weeks old) were housed in a temperature‐controlled environment (22 ± 1°C) with a 12‐h light/dark cycle. After a one‐week acclimatization, mice were randomly assigned to either a normal diet (ND, 10% kcal from fat; Research Diets D12450B) or a high‐fat diet (HFD, 60% kcal from fat; Research Diets D12492) for 20 weeks with ad libitum access to food and water. Body weight and food intake were monitored weekly. At week 22, mice were euthanized under deep anesthesia. Plasma, adipose depots (eWAT and sWAT), and major organs (heart, liver, spleen, lung, kidney, muscle, and brain) were harvested, weighed, and snap‐frozen in liquid nitrogen for storage at −80°C.

### 4.6 Plasmid Construction and Transfection Methods

The ER‐BioID2 (KDEL‐tagged), Golgi‐BioID2 (eNOS^1‐33^‐tagged), and bicistronic E/G‐BioID2 (IRES‐separated) expression plasmids were kindly provided by Prof. Li Wang (City University of Hong Kong). HEK293T and A549 cells (obtained from ATCC) were seeded in 6‐well plates at a density of 2 × 10^5^ cells/well 24 h prior to transfection. At 70%–80% confluency, cells were transfected with 4 µg of plasmid DNA using Lipofectamine 3000 (Invitrogen) in Opti‐MEM (Gibco) according to the manufacturer's instructions. The culture medium was replaced with complete DMEM after 6 h. Cells were harvested or utilized for downstream assays 48 h post‐transfection.

### 4.7 In Vitro Proximity Labeling

At 36–48 h post‐transfection, the culture medium was replaced with DMEM supplemented with 50 µm biotin (diluted from a 50 mM aqueous stock solution). Cells were incubated at 37°C with 5% CO_2_ for an additional 16 h to facilitate BioID2‐mediated protein biotinylation. Control groups were treated with medium lacking biotin. To ensure data consistency, cell morphology and viability were monitored throughout the labeling period. For the analysis of the culture supernatants (secretome), cells were meticulously washed three times with pre‐warmed PBS prior to biotin addition to minimize contamination from fetal bovine serum (FBS) proteins.

### 4.8 Protein Extraction and Preprocessing

Following the 16 h biotin labeling period, the culture supernatants were harvested and centrifuged at 1000 g for 10 min at 4°C to remove detached cells and cellular debris. The clarified supernatants were further concentrated and desalted using Zeba Spin Desalting Columns (7K MWCO, Thermo Fisher Scientific, Cat# 89877) according to the manufacturer's protocol. Briefly, columns were equilibrated with PBS, and the protein samples were processed via centrifugal filtration to remove excess free biotin and small molecular weight contaminants. For the cellular fraction, adherent cells were washed twice with ice‐cold PBS and lysed in RIPA buffer supplemented with a protease inhibitor cocktail. Both secretome and cellular protein extracts were quantified using a BCA assay, aliquoted, and stored at −80°C for downstream affinity purification and mass spectrometry analysis.

### 4.9 Protein Precipitation and Solubilization

To concentrate the secreted proteins and remove interfering substances, the collected supernatants were subjected to cold acetone precipitation. Briefly, four volumes of ice‐cold acetone were added to the samples, followed by incubation at −20°C for at least 1 h. The precipitated proteins were recovered by centrifugation at 12,000 g for 15 min at 4°C. The resulting pellets were washed once with cold acetone, air‐dried briefly at room temperature, and resuspended in RIPA lysis buffer. Both the resuspended secretome pellets and the adherent cell lysates were subjected to probe sonication (10% power, 2 s on/2 s off, for 2 min) on ice to ensure complete solubilization. The lysates were then clarified by centrifugation at 12,000 g for 15 min at 4°C. Total protein concentrations were determined using a BCA Protein Assay Kit (Thermo Fisher Scientific) according to the manufacturer's instructions, with absorbance measured at 562 nm using a microplate reader.

### 4.10 Affinity Purification of Biotinylated Proteins

Biotinylated proteins were enriched using Streptavidin Magnetic Beads (Pierce, Thermo Fisher Scientific, Cat# 88817). For each sample, the clarified protein lysates were incubated with pre‐washed streptavidin magnetic beads on a rotating wheel at 4°C overnight to ensure maximal binding. After incubation, the beads were captured using a magnetic rack, and the flow‐through was collected to monitor binding efficiency. To remove non‐specifically bound proteins, the beads were washed four times with Wash Buffer (PBS, pH 7.4, containing 0.05% Tween‐20). The beads were then washed twice with PBS to remove detergent traces prior to on‐bead digestion.

### 4.11 On‐Bead Proteolysis and Peptide Desalting

For label‐free quantitative analysis, the protein‐bound beads were subjected to on‐bead digestion. Briefly, beads were resuspended in 50 µL of Elution Buffer I (2 m urea, 50 mM Tris‐HCl pH 8.0, and 1 mm DTT) supplemented with 0.5 µg of sequencing‐grade trypsin (Thermo Fisher Scientific, Cat# 90057). The mixture was incubated at 30°C for 60 min with shaking at 700 rpm. The supernatant was collected, and the beads were further washed twice with 25 µL of Elution Buffer II (2 m urea, 50 mm Tris‐HCl pH 8.0, and 5 mm iodoacetamide). All eluates were pooled and further digested with an additional 250 ng of trypsin at 32°C overnight in the dark. The reaction was stopped by adding 10% formic acid (v/v, 1:25). The resulting peptides were desalted using C18 StageTips (Thermo Fisher Scientific, Cat# 87784) according to the manufacturer's instructions. Samples were concentrated using a SpeedVac vacuum concentrator and stored at ‐80°C prior to LC‐MS/MS analysis.

### 4.12 LC‐MS/MS Analysis and Data Processing

Desalted peptides were reconstituted in 0.1% (v/v) formic acid (FA) and analyzed on an Easy‐nLC 1200 system coupled to a Q‐Exactive mass spectrometer via an Easy‐Spray ion source (Thermo Fisher Scientific). Peptides were separated on a C18 analytical column (75 µm i.d. × 250 mm) using a 75‐min linear gradient of 3%–95% acetonitrile in 0.1% FA at a flow rate of 300 nL/min. The mass spectrometer operated in data‐dependent acquisition (DDA) mode. Full MS scans were acquired in the Orbitrap (350‐1800 m/z) at a resolution of 120,000, and the top 15 most intense precursor ions were selected for HCD fragmentation (NCE of 35%) with MS2 spectra acquired at a resolution of 30,000. Dynamic exclusion was set to 15 s. Raw MS data were processed using Proteome Discoverer 2.2 with the Sequest HT search engine against the respective UniProt reference proteomes (Homo sapiens or Mus musculus). Search parameters included: trypsin digestion with up to two missed cleavages; carbamidomethylation (C) as a fixed modification; and oxidation (M), deamidation (N/Q), and protein N‐terminal acetylation as variable modifications. Precursor and fragment mass tolerances were set to 10 ppm and 20 ppm, respectively, with both protein and peptide false discovery rates (FDR) filtered at <1%. Label‐free quantification (LFQ) was performed using the Minora Feature Detector algorithm. For comparative analysis, protein abundances were log2‐transformed and statistically evaluated using the limma package in R.

### 4.13 Immunofluorescence Microscopy

Cells were seeded onto glass coverslips in 6‐well plates and grown to 50%–70% confluency. After washing with PBS, cells were simultaneously fixed and permeabilized with 4% paraformaldehyde containing 0.2% (v/v) Triton X‐100 for 30 min at room temperature. After three washes with PBS containing 2% BSA (5 min each), cells were blocked with 2% BSA for 15 min. To verify the organelle localization of BioID fusion proteins and biotinylated targets, cells were probed with a mouse anti‐HA‐Tag antibody (Santa Cruz Biotechnology, sc‐7392) and an organelle‐specific rabbit primary antibody, which included anti‐TGN46 (Proteintech, 13573‐1‐AP) or anti‐Calnexin (Thermo Fisher Scientific, PA5‐34754). A rabbit anti‐GM130 antibody (Thermo Fisher Scientific, PA1‐077) was used for tissue samples. Primary antibodies were incubated in a humidified chamber (diluted 1:100–1:500 in 2% BSA) at room temperature for 45 min or overnight at 4°C. Following three washes, target proteins were labeled with fluorophore‐conjugated secondary antibodies and streptavidin (diluted 1:200–1:500 in 2% BSA) for 1h in the dark at room temperature. The detection reagents included Goat anti‐Rabbit IgG Alexa Fluor 488 (Thermo Fisher Scientific, A‐11008), Donkey anti‐Mouse IgG Alexa Fluor 594 (Thermo Fisher Scientific, A‐21203), and Alexa Fluor 647‐conjugated Streptavidin (Invitrogen). Nuclei were counterstained with DAPI (1 µg/mL) for 10 min. Finally, coverslips were mounted onto glass slides using an anti‐fade mounting medium. Confocal images were acquired on a Nikon A1R HD25 inverted microscope with a 20X objective (NA 0.75) using NIS‐Elements Imaging software and subsequently processed using FIJI.

### 4.14 Preparation and Purification of Biotinylated Leptin

Biotinylation of recombinant leptin was performed using Biotin‐7‐NHS ester. Briefly, the Biotin‐7‐NHS powder was reconstituted in anhydrous DMSO to a stock concentration of 20 mg/mL. Leptin (1 mg) was dissolved in 1 mL of PBS (pH 7.4), and the biotin reagent was added at a 5:1 molar ratio (biotin:protein), ensuring the DMSO content remained below 5% (v/v) to preserve protein stability. The conjugation reaction was allowed to proceed for 2 h at room temperature (15°C–25°C) with continuous mild agitation. To remove unreacted biotin, the reaction mixture was subjected to size‐exclusion chromatography using a Sephadex G‐25 column. The column was pre‐equilibrated with 5 mL of blocking solution followed by 30 mL of PBS. The labeled leptin was eluted with PBS, and fractions were monitored by UV absorbance at 280 nm. The protein‐rich fractions were pooled and concentrated to a final range of 1–5 mg/mL using a 10 kDa molecular weight cut‐off (MWCO) ultrafiltration centrifugal filter. For long‐term storage, the biotinylated leptin was supplemented with 1% BSA as a stabilizer and stored at −20°C.

### 4.15 In Situ BSPA Assay

Frozen sections were thawed and fixed with 4% paraformaldehyde, followed by permeabilization with 0.5% Triton X‐100. After blocking with 3% BSA, sections were incubated with 50 µm probeA (5’‐azide‐AAAAA AAAAT ATGAC AGAAC TAGAC ACTCT T‐3’) conjugated to anti‐leptin antibodies (R&D systems, AF498), then treated with streptavidin at 25°C. For the ligation‐mediated assembly, a hybridization mixture was applied containing 1 µM biotin‐labeled probeB (5’‐GACG CTAA TAGT TAAG ACGC TTTA ATCG TGGA TCAG TCTA GACAT GTCACTAACATCATCATTC AAAGTTCTCGA ATCATGTATC ACGCTGTA‐biotin‐3’) and auxiliary oligonucleotides, including Circle‐1 (5’‐Phosphate‐CTATT AGCGT CCAGT GAATG AACTA TACAA CATAC TACCT CAGCC GTCAA GAGTG TCTA‐3’) and Circle‐2 (5’‐Phosphate‐GTTCT GTCAT ATTTA AGCGT CTTAA‐3’). Subsequent circularization was conducted at 37°C for 2 h using 0.5 U/µL T4 DNA ligase. The rolling circle amplification (RCA) was then performed at 37°C for 3 h using 0.5 U/µL phi29 DNA polymerase and 1 mM dNTPs. The resulting RCA products were visualized by hybridizing with 100 nM DP‐488 (5’‐Alexa488‐AACTA TACAA CATAC TACCT CA‐3’) at 37°C for 30 min. Following nuclear counterstaining with DAPI, the slides were mounted and imaged using confocal microscopy. Fluorescence quantification of BSPA signals was performed using Fiji software. To ensure precise measurement and proper signal normalization, representative confocal images from mouse brain sections were analyzed. For each condition, multiple regions of interest (ROIs) were defined. The mean fluorescence intensity (MFI) within each ROI was measured. To eliminate tissue autofluorescence and instrumental noise, local background fluorescence from an adjacent signal‐free area was subtracted from the MFI of each target ROI. The specific sample size (n) for each experiment is indicated in the respective figure legends, with three slides analyzed per mouse. All image acquisition settings, including laser power, gain, and pinhole, were maintained strictly identical across all experimental groups.

### 4.16 Stereotaxic Microinjection and Laser Capture Microdissection

Mice were anaesthetised and secured on a digital mouse stereotaxic instrument (Stoelting, Wood Dale, IL, USA). Bilateral microinjections were performed using a glass micropipette connected to a Nanoject III system (Drummond Scientific, Broomall, PA, USA) at coordinates (relative to bregma): AP ‐1.55 mm, ML ±3.5 mm, and DV ‐5.0 mm. Mice received a unilateral injection of recombinant leptin (400 ng/µL, 0.6 µL) into the right hemisphere, while the left hemisphere was injected with an equal volume of endotoxin‐free 1× PBS as a vehicle control. The injection flow rate was 0.1 µL/min. The micropipette was left in place for 10 min post‐injection before slow withdrawal. The scalp was sealed with tissue adhesive glue (3M, St. Paul, MN, USA), and mice were allowed to recover on a heating pad. Two hours post‐injection, the mice were sacrificed to harvest tissues for subsequent laser capture microdissection. Cryopreserved tissue blocks were equilibrated at −19°C in a cryostat chamber for 30 min prior to sectioning. Tissue sections were stained with 1% (w/v) cresyl violet (filtered through a 0.22 µm syringe filter) for 1 min at room temperature. For dehydration and destaining, slides were sequentially immersed in 75% ethanol for 3 s, 95% ethanol for 5 s, and 100% ethanol for 10 s. Air‐dried slides were stored in a desiccated environment on ice to maintain RNA integrity. Before microdissection, samples were equilibrated to ambient temperature for 15 min to prevent moisture condensation. Laser microdissection was performed using a Leica LMD system at 20x magnification. Laser parameters were optimized as follows: power, 55; aperture, 1; speed, 4; bridge size, 7; final pulse, 23; head current, 73%; and pulse frequency, 174 Hz. Targeted areas were precisely demarcated and collected into 0.5 mL microcentrifuge tubes. Following dissection, tubes were briefly centrifuged at 500 g for 10 s to pellet the harvested tissue. For RNA extraction, 350 µL of RLT lysis buffer was added to the collected samples. To ensure maximum recovery, the “Move+Cut” function was utilized for any residual tissue fragments. Total RNA was extracted from the microdissected tissues using RNeasy Micro Kit (Qiagen, Cat. No. 74004). RNA concentration and integrity were assessed using an Agilent 2100 Bioanalyzer (Agilent Technologies) followed by subsequent high‐throughput sequencing. Besides, for RT‐qPCR experiments, the expression level of target genes was normalized to that of β‐actin (Actb), and the relative fold change in mRNA expression was calculated using the 2^−ΔΔCt^ method.

### 4.17 Functional Enrichment and Network Analysis

Gene Ontology (GO) enrichment analysis was conducted using the DAVID bioinformatics resources (https://davidbioinformatics.nih.gov/). Statistical significance was determined using the Benjamini‐Hochberg procedure to adjust p‐values for multiple hypothesis testing. Enriched GO terms within the Biological Process (BP) and Cellular Component (CC) categories were selected based on a False Discovery Rate (FDR) threshold of < 0.05. The top ten most significantly enriched terms were utilized for covariation analysis and gene colocalization interrogation unless otherwise specified. Functional redundancy and interrelationships between the enriched terms were visualized as an enrichment network using the EnrichmentMap plugin within Cytoscape.

### 4.18 Quantification and Statistical Analysis

Statistical analyses were performed using GraphPad Prism 9. Data are presented as the mean ± standard deviation (SD). For pairwise comparisons, statistical significance was determined using a two‐tailed Student's *t*‐test, while multiple‐group comparisons were evaluated via one‐way analysis of variance (ANOVA). A 95% confidence interval was applied to all statistical tests, with significance defined as *p* < 0.05. Sample sizes (*n*) were determined based on established standards in the field for specific experiment types. All replicates cited in this study refer to biological replicates unless otherwise stated. For in vivo studies, mice were randomly assigned to experimental groups. Quantitative immunoblot data represent the mean values from 2–3 independent experiments. For mass spectrometry‐based proteomics, samples were processed in a randomized order, and investigators were blinded to the experimental conditions during data acquisition and initial analysis. The exact values of *n*, denoting the number of mice or independent biological replicates, are specified in the respective figure legends.

## Author Contributions

L.Z. conceived and designed the study. F.Y. and R.Q. conducted the experiments and analyzed the data. L.W., J.Y.K and K.O.L. provided technical support and supervision. J.S. performed the cultivation of neuronal cells. F.Y., R.Q. and X.M performed the stereotaxic injection experiment. F.Y. and R.Q. prepared the figures and drafted the manuscript. L.Z. and L.W. edited and revised the manuscript. L.Z. supervised the project and approved the final manuscript.

## Conflicts of Interest

The authors declare no conflicts of interest.

## Supporting information




**Supporting File 1**: advs77190‐sup‐0001‐SuppMat.docx.

**Supporting File 2**: advs77190‐sup‐0002‐TableS1.xlsx.

**Supporting File 3**: advs77190‐sup‐0003‐TableS2.xlsx.

**Supporting File 4**: advs77190‐sup‐0004‐TableS3.csv.

**Supporting File 5**: advs77190‐sup‐0005‐TableS4.csv.

**Supporting File 6**: advs77190‐sup‐0006‐TableS5.csv.

## Data Availability

The proteomics datasets generated in this study have been deposited in iProX (accession number PXD075801). The sequencing data have been deposited in NCBI BioProject (accession number PRJNA1401511). Processed proteomics datasheets used for all main and supplementary analyses are included in Table S1–S5. Any additional information or materials required to reanalyze the data reported in this study are available from the lead contact upon reasonable request.
