# Supplementary material for: Dual‐Organelle Secretome Conjugation and Organ‐Uptake Tracking (DuO‐SCOUT) Enables Deep and Sensitive Mapping of the Secreted Proteins
Source: Adv Sci (Weinh). 2026 Aug 14:e77190. Online ahead of print. doi: 10.1002/advs.77190 (PMC13474175; doi:10.1002/advs.77190)
Supplement: Supplementary file 1 [file ADVS-9999-e77190-s004.docx]

**Supplemental information**

**Dual-Organelle Secretome Conjugation and Organ-Uptake Tracking (DuO-SCOUT) Enables Deep and Sensitive Mapping of the Secreted Proteins**

Fenglian Yang^1,2,4^, Rui Qian^1,2,4^, Jiaxing Song^3^, Xingqi Meng^1^, Jin Young Kim^1^, Kwok On Lai^3^, Li Wang^1,2*^ and Liang Zhang^1,2*^

^1^Department of Biomedical Sciences, College of Biomedicine, City University of Hong Kong, Hong Kong SAR, China.

^2^Biotech and Health Centre, Shenzhen Research Institute of City University of Hong Kong, Shenzhen, Guangdong, China.

^3^Department of Neuroscience, College of Biomedicine, City University of Hong Kong, Hong Kong SAR, China.

^4^These authors contributed equally

Address correspondence to:

*Liang Zhang, [liangzhang.28@cityu.edu.hk](mailto:liangzhang.28@cityu.edu.hk)

*Li Wang, [li.wang@cityu.edu.hk](mailto:li.wang@cityu.edu.hk)

Figure S1. (A-B) Immunoblotting analysis of biotinylated proteins in (A) cell lysates and (B) culture supernatants of A549 cells transfected with control vector, ER-IRES-Golgi-BioID2 complex (bicistronic), or ER-BioID2 co-transfected with Golgi-BioID2 (co-transfection).

## Figure S2. Activation of E/G BioID2 in Adipose Cre+ mice.

## (A) PCR identification results of target genes. The left panel shows the cre+ mice Adipoq-cre^+/-^ (two bands) and the cre- mice Adipoq-cre-/- (one band); the right panel shows the flox+ mice E/G-BioID2^flox/flox^ (two bands).

## (B) Western blot identification of biotinylated proteins in Adipoq-cre^+/-^-E/G-BioID2^flox/flox^ mice. This analysis presents the expression levels of biotinylated proteins across different tissues, highlighting variations in biotinylation.

Figure S3. Comparison of weight change trend of mice between ND and HFD group**.**

Figure S4. Comparison of cre+ and cre- group between ND and HFD group in plasma. Error bars, standard deviation. *n* = 4 mice per condition. *t*-test, **p* <0.05, ***p* <0.01**.**

Figure S5. Quantification of PLA signals between ND and HFD group in mouse brain.

Figure S6. Local administration of recombinant leptin in the piriform cortex promotes Il6 mRNA transcription. (A) Schematic illustration of the injection of recombinant leptin into the piriform cortex. (B) Representative confocal micrographs of brain slices showing the localization of injected leptin (Green). Nuclei are counterstained with DAPI (Blue).

Figure S7. Lack of colocalization between bioPLA signals and CD31+ brain vasculature. Immunofluorescence staining of bioPLA (green) and CD31 (pink) confirms that the biotinylated leptin is non-vascular origin of the signals.

## Figure S8. Agilent 2100 Bioanalyzer gel image of isolated total RNA from microtissues.

The main peaks of 28S rRNA (approximately 4,000 nucleotides) and 18S rRNA (approximately 1,800 nucleotides) were clear and intact, with no significant degradation or contamination, indicating that the RNA samples met the requirements for transcriptome sequencing and were of high quality for subsequent library construction and sequencing.

Figure S9. Clustering analysis of gene expression data between HFD group and ND group mouse in (A) HT regions and (B) PIR regions.
